# Increased Global-Brain Functional Connectivity Is Associated with Dyslipidemia and Cognitive Impairment in First-Episode, Drug-Naive Patients with Bipolar Disorder

**DOI:** 10.1155/2021/5560453

**Published:** 2021-06-05

**Authors:** Pan Pan, Yan Qiu, Ziwei Teng, Sujuan Li, Jing Huang, Hui Xiang, Hui Tang, Jindong Chen, Chujun Wu, Kun Jin, Bolun Wang, Feng Liu, Haishan Wu, Wenbin Guo

**Affiliations:** ^1^National Clinical Research Center on Mental Disorders and Department of Psychiatry, The Second Xiangya Hospital of Central South University, Changsha, Hunan 410011, China; ^2^Department of Radiology, The Second Xiangya Hospital of Central South University, Changsha, Hunan 410011, China; ^3^Department of Radiology, Tianjin Medical University General Hospital, Tianjin 300000, China; ^4^Department of Psychiatry, The Third People's Hospital of Foshan, Foshan, Guangdong 528000, China

## Abstract

**Objectives:**

Previous researches have demonstrated that abnormal functional connectivity (FC) is associated with the pathophysiology of bipolar disorder (BD). However, inconsistent results were obtained due to different selections of regions of interest in previous researches. This study is aimed at examining voxel-wise brain-wide functional connectivity (FC) alterations in the first-episode, drug-naive patient with BD in an unbiased way.

**Methods:**

A total of 35 patients with BD and 37 age-, sex-, and education-matched healthy controls underwent resting-state functional magnetic resonance imaging (rs-fMRI). Global-brain FC (GFC) was applied to analyze the image data. Support vector machine (SVM) was adopted to probe whether GFC abnormalities could be used to identify the patients from the controls.

**Results:**

Patients with BD exhibited increased GFC in the left inferior frontal gyrus (LIFG), pars triangularis and left precuneus (PCu)/superior occipital gyrus (SOG). The left PCu belongs to the default mode network (DMN). Furthermore, increased GFC in the LIFG, pars triangularis was positively correlated with the triglycerides (TG) and low-density lipoprotein cholesterol (LDL-C) and negatively correlated with the scores of the Repeatable Battery for the Assessment of Neuropsychological Status (RBANS) coding test and Stroop color. Increased GFC values in the left PCu/SOG can be applied to discriminate patients from controls with preferable sensitivity (80.00%), specificity (75.68%), and accuracy (77.78%).

**Conclusions:**

This study found increased GFC in the brain regions of DMN; LIFG, pars triangularis; and LSOG, which was associated with dyslipidemia and cognitive impairment in patients with BD. Moreover, increased GFC values in the left PCu/SOG may be utilized as a potential biomarker to differentiate patients with BD from controls.

## 1. Introduction

Bipolar disorder (BD) is a severe and chronic mood disorder characterized by alternating episodes of depression and mania, punctuated by periods of clinical remission or euthymia [1]. The prevalence rate of BD is relatively high (0.5%-1.5%) [2, 3]. The high mortality of patients with BD is caused by cardiovascular diseases, other natural causes, and suicide [4, 5]. Patients with BD who are associated with metabolic syndrome have complex clinical presentations, difficult treatment, and increased risk of suicide. BD is currently diagnosed by observing patients' behavior and self-reporting. Therefore, objective biomarkers are needed to improve the early recognition rate and the diagnosis rate of BD [6].

Metabolic syndrome is a common complication in patients with BD. In particular, dyslipidemia is a risk factor for the onset of cardiovascular disease which is often manifested by elevated triglycerides (TG), total cholesterol, high-density lipoprotein cholesterol (HDL-C), and low-density lipoprotein cholesterol (LDL-C) in the serum. The prevalence of dyslipidemia in patients with BD could be as high as 37.3% [7]. The lowest rate of dyslipidemia (hypertriglyceridemia or low HDL-C) was observed in emergent mania [8, 9]. Different from other mood disorders, BD may be accompanied by state-dependent low cholesterol and TG levels [7]. Therefore, exploring the association between dyslipidemic levels and the severity of BD is warranted.

Cognitive impairment is a recognized characteristic of mood disorders which is common in BD. Patients with BD show dysfunction in several cognitive areas including attention, executive function, learning and memory, and psychomotor speed, during acute episodes of mania and depression [10]. Persistent cognitive deficits could be found in roughly one-third of patients with BD during euthymia after the major emotional symptoms were resolved [11]. One research suggested that cognitive impairment was an important reason for the inability to restore social function in patients with euthymia [12]. Neuroimaging studies of mood disorders have proven that cognitive impairment originates from the destruction of neuroplasticity mechanisms and the functional and structural changes of cognition-related neural circuits [13]. However, the specific neurobiological mechanisms associated with cognitive impairment remain unclear.

Resting-state functional connectivity (FC) between brain regions is an essential tool for understanding FC alterations between brain regions in mental disorders. Previous studies focused on using a region-of-interest (ROI) method to study the alterations of FC in preselected brain regions with inconsistent results [14–20]. The reason is that these studies may ignore the key areas related to the core pathological alterations in BD due to the preselected ROIs. Different ROI selections may yield different results due to potentially biased results based on preset ROIs. Voxel-based global-brain FC (GFC) analysis can be adopted to probe the pathophysiology of BD to remedy this defect. GFC is a measurement that focuses on FC alterations throughout the whole brain rather than the preselected ROIs [21, 22]. The advantage relative to other FC approaches is that GFC can reflect the complexity of the whole brain connectome. This method can avoid parcellation-dependent effects on the topological organization of the brain network [23]. GFC focuses on the relationship of a given voxel to all other voxels of the brain, not just its relationship to a single region or to separate larger components [24]. Given this background, the purpose of the GFC method adopted in the study was to observe brain mechanism from the perspective of FC alterations across the whole brain in an unbiased way.

As a monitoring machine learning technology, the support vector machine (SVM) has been applied to medical diagnosis and image processing. SVM is a computational algorithm that learns from experience and examples to assign labels to targets. Its basic function is to separate binary labeled data based on a line to maximize the distance between the labeled data [25]. SVM has good accuracy under limited samples [26]. The present study was aimed at exploring FC alterations in the whole brain of patients with BD by using the GFC method. Based on the aforementioned studies in BD, we hypothesized that (1) patients with BD would exhibit significant GFC alterations in certain brain regions compared with healthy controls, (2) abnormal GFC in these brain regions might be associated with blood lipid levels, and (3) abnormal GFC values could be utilized as potential biomarkers to differentiate patients with BD from healthy controls.

## 2. Materials and Methods

### 2.1. Subject

A total of 38 right-handed patients with BD aged 16-45 years old were recruited from the Second Xiangya Hospital of Central South University. All patients were diagnosed and screened by two experienced psychiatrists based on the Diagnostic and Statistical Manual of Mental Disorders, Fifth Edition (DSM-5). The enrolled patients were first-episode patients without any drug treatment or psychotherapy to their BD and with the course of disease not exceeding 5 years. Data on clinical characteristics were collected through direct interviews with patients and their relatives as well as access to the patients' medical records. The exclusion criteria included any serious organic disease and other mental disorders in accordance with DSM-5, any history of alcohol or drug abuse, and lipid-lowering treatment, pregnancy, and contraindications for MRI scan. We used the Hamilton Depression Rating Scale-17 (HAMD-17), Young Mania Rating Scale (YMRS), and Hamilton Anxiety Scale-14 (HAMA-14) to assess the clinical symptoms of BD. Repeatable Battery for the Assessment of Neuropsychological Status (RBANS) was adopted to evaluate the cognitive functions of all patients.

Forty right-handed healthy controls were recruited through advertising in the local community at the same time. All controls were matched with patients in age, sex ratio, and education. Healthy controls were screened using DSM-5, nonpatient version. None of the controls or their first-degree relatives had any history of serious mental disease, neurological disease, or substance abuse.

The study was approved by the ethics committee of the Second Xiangya Hospital of Central South University and was performed in accordance with the Helsinki Declaration. All participants provided a written informed consent after a complete explanation. A parental consent was obtained for participants under 18 years old.

### 2.2. Sample Collection

Fasting blood samples from all patients were collected between 7 am and 9 am for biochemical analysis to avoid circadian disruptions of the data. Serum test was adopted to analyze the following parameters: liver and kidney function, blood glucose, TG, HDL-C, and LDL-C.

### 2.3. Image Acquisition and Preprocessing

Resting-state images were obtained using a Siemens 3.0T scanner, and the imaging data were preprocessed automatically using the DPABI software in MATLAB [27]. Participants were instructed to lie motionless during the scan. Detailed image acquisition and preprocessing procedures are provided in the supplementary file (available here).

### 2.4. GFC Analysis

GFC is a data-driven graph theory approach that measures the number of FCs between a given voxel and other voxels within a gray matter mask [28]. The gray matter mask is produced by the gray matter probability map (Probability > 0.2) in SPM8 [29]. Such a threshold was chosen to eliminate the voxel with weak correlations that possibly originated from signal noise [29]. GFC of a given voxel was defined as the mean Pearson coefficients (*r*) between the time series of this voxel with all other voxels within the gray matter mask. We computed the mean correlation coefficients throughout the gray matter mask in the whole brain in the MATLAB [21, 30–33]. All correlation coefficients were considered for the average, including positive and negative values. The Fisher *r*-to-*z* transformation was utilized to convert the coefficients into *z* values to improve data normality [34–36]. The GFC maps were generated by composing GFC of all voxels within the gray matter mask. This method has been applied in several neuropsychiatric disorders by our group, including schizophrenia, somatization disorder, cervical dystonia, dry eye disease, primary blepharospasm, obsessive-compulsive disorder, and major depressive disorder [21, 32, 37–41].

### 2.5. Classification Analysis

SVM is a good learning classifier widely used in classification, especially for small sample cases [42, 43]. The basic function of SVM is to separate binary labeled data based on a line to maximize the distance between labeled data [44]. It learns from experience and examples on how to assign labels to targets and uses kernel functions to separate labeled data. One advantage of using the kernel function in SVM is that it could be applied to nonvector inputs, which is important in the medical field [26, 44, 45]. The kernel type used in this study was the default Gaussian kernel in MATLAB. The LIBSVM software package in MATLAB [46] was employed in this study. Abnormal clusters were obtained from the group comparisons between patients and controls. We then extracted mean *z* values from brain clusters with abnormal GFC. The sample set was divided into a test set and a training set to observe the classification performance of label data [47]. A random SVM was established to classify and select the brain clusters based on the fMRI data obtained from the brains of the participants. A “leave-one-out” cross-validation method was utilized to optimize the parameters, and the most common traits were selected to obtain better sensitivity and specificity.

### 2.6. Statistical Analysis

Continuous variables, including age and years of education, were analyzed with two-sample *t*-tests. A chi-squared test was used for sex distribution. The significance level was set at *p* < 0.05.

Two-sample *t*-tests were adopted to compare GFC differences between patients and controls. We calculated the frame-wise displacement (FD) values of each participant based on a previous research [48]. The education level, sex, age, and mean FD were treated as uninterested covariates. The family-wise error (FWE) correction method was adopted to set the significance level at *p* < 0.05.

Pearson correlation analyses were conducted between GFC values of BD and clinical variables including blood lipid level and scale scores of HAMD-17, HAMA-14, YMRS, and RBANS after extracting the mean *z* values of GFC from the brain clusters with abnormal GFC and evaluating the normalization of the conversion values. The significance level was Bonferroni corrected at *p* < 0.05.

## 3. Results

### 3.1. Characteristics of the Participants

We excluded three patients and three controls before further investigation due to excessive head movement during fMRI scanning. The final sample included 35 patients with BD and 37 healthy controls. No significant differences were observed in sex ratio, age, and years of education between patients with BD and controls. The clinical variable characteristics of the participants are shown in Table 1. The detailed results with each value for male and female are shown in supplementary Table S1.

### 3.2. Group Differences in GFC

**Figure 1:**
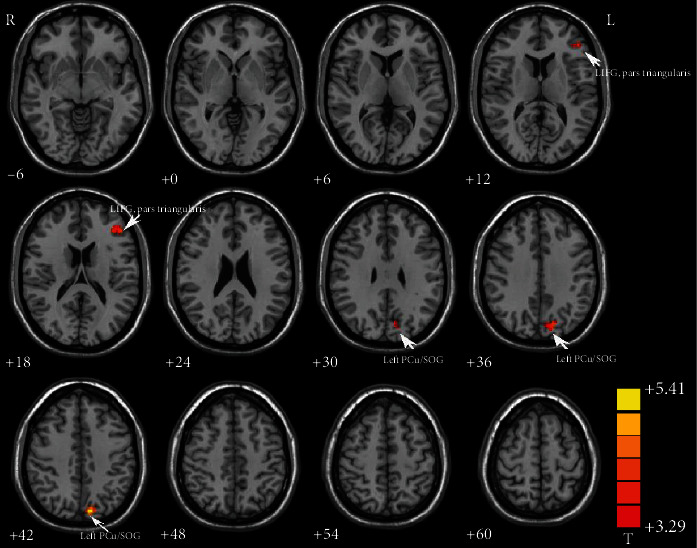
Increased GFC in the LIFG, pars triangularis (*t* = 4.1653) and left PCu/SOG (*t* = 5.3697) in patients with BD relative to healthy controls. GFC: global-brain functional connectivity; LIFG: left inferior frontal gyrus; PCu: precuneus; SOG: superior occipital gyrus; BD: bipolar disorder.

Compared with healthy controls, increased GFC in the left inferior frontal gyrus (LIFG), pars triangularis (*t* = 4.1653, *p* < 0.001) and left precuneus (PCu)/superior occipital gyrus (SOG) (*t* = 5.3697, *p* < 0.001) was observed in patients with BD (Figure 1 and Table 2). No decreased GFC was found in any brain regions in the patients.

### 3.3. Correlations between GFC and Clinical Variables

**Figure 2:**
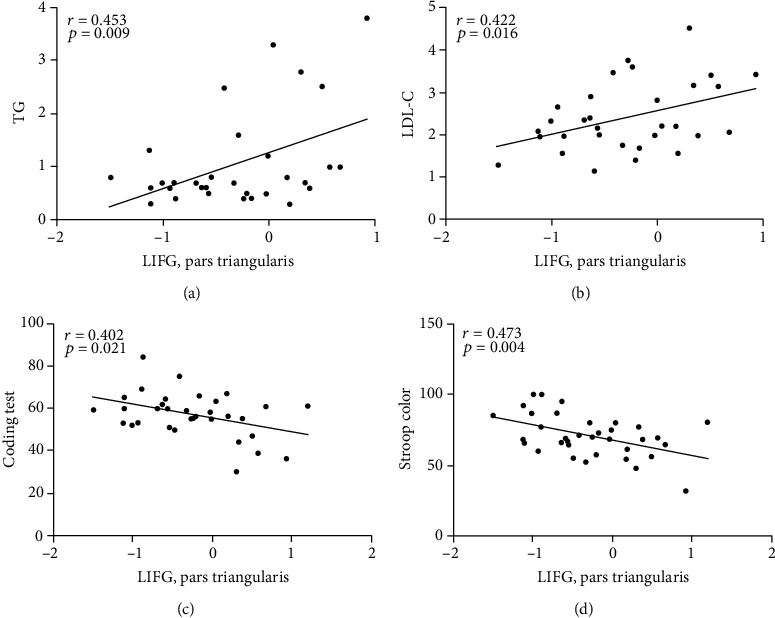
(a, b) Positive correlations between the GFC values in the LIFG, pars triangularis and the TG or LDL-C in patients with BD. (c, d) Negative correlations between the GFC values in the LIFG, pars triangularis and Stroop color or coding test of the RBANS in patients with BD. BD: bipolar disorder; GFC: global-brain functional connectivity; LIFG: left inferior frontal gyrus; RBANS: repeatable battery neuropsychological status; TG: triglycerides; LDL-C: low-density lipoprotein cholesterol.

As shown in Figure 2, GFC values in the LIFG, pars triangularis were positively correlated with the TG (*r* = 0.453, *p* = 0.009) and LDL-C (*r* = 0.422, *p* = 0.016) and negatively correlated with the scores of the RBANS coding test (*r* = −0.402, *p* = 0.021) and Stroop color (*r* = −0.473, *p* = 0.004). No significant correlation was found between the GFC values and the illness duration, years of education, age, and scores of HAMD-17, HAMA-14, or YAMS.

### 3.4. SVM Results

**Figure 3:**
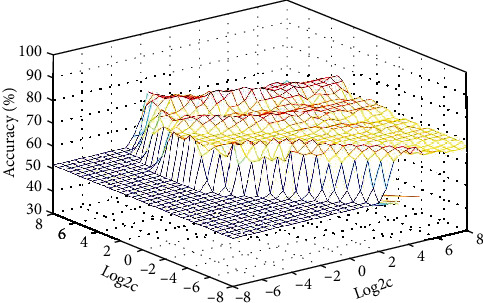
3D view of classified accuracy with the best parameters. Visualization of classifications in SVM by using increased GFC values in the abnormal brain region. The result was obtained in LIBSVM using a “leave-one-out” approach with default Gaussian kernel. GFC: global-brain functional connectivity; SVM: support vector machines.

Increased GFC values in the left PCu/SOG could identify the patients with BD from the controls with preferable sensitivity (80.00%), specificity (75.68%), and accuracy (77.78%) (Table 3 and Figure 3). As shown in Table 3, the accuracy of LIFG was unsatisfactory.

## 4. Discussion

Increased GFC in the LIFG, pars triangularis and left PCu/SOG was observed in patients with BD relative to controls, indicating disrupted functional interactions in the DMN, LSOG, and LIFG, pars triangularis in the patients. The result of SVM analysis suggested that increased GFC in the left PCu/SOG could differentiate patients with BD from controls with preferable sensitivity and accuracy. Moreover, GFC values in the LIFG, pars triangularis were positively correlated with the TG, LDL-C and negatively correlated with the scores of the RBANS coding test and Stroop color.

The IFG is located below the subfrontal sulcus. The IFG, pars triangularis is in the middle of the IFG. Increased FC in the brain region was considered as a compensatory effort for the brain activation in the resting state [49, 50]. Compared with healthy controls, patients with BD exhibited increased GFC in the LIFG, pars triangularis, which might represent increased cortical thickness in this area and/or an inflammatory response at the early stage of the disease because patients with BD were first-episode patients without any drug treatment or psychotherapy to their BD and with the course of disease not exceeding 5 years in the present study. At this stage, astrocytes, which make up the majority of cortical tissue, can be activated by proinflammatory cytokines [51] and lead to cellular hyperplasia, hypertrophy, and increased cortical thickness [52]. Activated astrocytes will stimulate neuronal survival by producing neurotrophic factors that will promote the recovery of central nervous system function. In addition, increased GFC of the LIFG, pars triangularis in patients with BD was negatively correlated with the scores of the RBANS coding test, suggesting a strong association with cognitive function of this region. Combined with previous studies, the present study indicated that compensation might occur in the triangular part.

The prefrontal lobe plays a key role in emotional and cognitive control [53]. Previous studies found abnormal neurobiochemistry and neuropathology in the prefrontal lobe in patients with BD. Moreover, the dysfunction in the prefrontal lobe was closely related to cognitive dysfunction in patients with affective disorder [54, 55]. The BA45 and 44 in the LIFG are Broca' s regions involved in language production [56] which provide a pathway for the BA47/12 in the lateral orbitofrontal cortex to connect to the premotor region. The LIFG, pars triangularis is the region connecting the language processing in the left hemisphere with the signal output from the premotor cortex region [57]. Thus, we speculated that the dysfunction in the LIFG, pars triangularis might explain the symptom of alexithymia during the manic state. Alexithymia is defined as an emotional experience with which an individual has difficulty in recognizing and expressing oneself and is widely regarded as an impairment in the process of emotional recognition, processing, and regulation [58]. Studies in BD have generally shown that patients with BD present a high prevalence of alexithymia compared to healthy controls [59, 60]. The manic state in patients with BD tends to be brief and exhibits significant alexithymia during these periods [61]. We are sorry that we did not assess alexithymia in the patients in this study because patients with BD included in the present study were at the depressive episode.

Patients with BD accompanied with alexithymia showed poor emotional regulation ability [62]. A previous study on coding task in patients with ischemic lesions speculated that the LIFG might be involved in coding behavior [63]. Consistent with that study, our result exhibited that the GFC values in the LIFG, pars triangularis were negatively correlated with the scores of the RBANS coding test. This result suggested that the LIFG might be one of the main areas involved in coding. The Stroop Color-Word Task was used to investigate response inhibition. In this study, the GFC values in the LIFG, pars triangularis were negatively correlated with the Stoop color score. Increased GFC in the LIFG of patients with BD and the controlling effect of response inhibition were emphasized in the color-word interference process which may reflect the defects in the executive control of patients with BD, especially the defects in response inhibition treatment [64].

The PCu is associated with several high-level cognitive functions and may be involved in information processing related to metacognitive processes, such as episodic memory, self-reflection, and other introspection [65]. A previous research found that the metacognitive process dysfunction of patients with BD in the social and emotional domains was closely associated with self-reflection and other reflection [66]. Several studies have shown that autobiographical memory may play a key role in the processing of self-reflection and other reflection [67]. The PCu is an important region that constitutes autobiographical memory [68, 69]. In other words, the PCu contributes specifically to self-reflection [67] and other reflection, which are related to autobiographical memory processing. Abnormal function in PCu of patients with BD might lead to impaired autobiographical memory and make them provide less details when recalling autobiographical memory compared with the controls [66]. The most sensitive brain network to resting state is the DMN. DMN is active when the brain is at rest and inactive when the brain is involved in task execution. The PCu is a crucial part of the DMN. As the functional core node of DMN [70], the PCu is the only hub to distinguish between the task and resting state of the brain [71]. As the only network node in the DMN that directly interacts with other nodes, PCu dysfunction may impair the connection to the entire DMN [72] and affect the ability of the brain to distinguish between task execution and resting state. Therefore, the present results suggested disrupted functional interactions in the DMN in the patients compared with the controls.

The LSOG is involved in the processing of advanced visual association and is an important part of the dorsolateral cortex [73]. Previous studies have exhibited that the SOG is activated in tasks related to emotional recognition [74–76] and may be associated with emotional processing [77]. Another study has shown that individuals with higher anxiety are more sensitive to sensory information and tend to pay attention to visual fear information [78]. Individuals with abnormal FC in the SOG may feel anxiety more easily than those with normal FC in the SOG [78]. The present study observed increased GFC in the LSOG of patients with BD. Thus, the difficulty of patients with BD to recognize emotions may be related to the processing of visual information.

The risk of comorbidity with metabolic syndrome characterized by dyslipidemia in BD is high (about 37%) [7]. The prevalence of hypertriglyceridemia in patients with BD is relatively high [79]. Notably, increased GFC in the LIFG, pars triangularis is positively correlated not only with TG but also with LDL-C. Previous studies have confirmed that reduced TG might be associated with suicidal and self-injurious behavior of depression or BD [80–82]. Patients with BD have persistent cognitive impairment in mood episodes which has not been recognized until now [83]. In addition, studies on the relationship between lipid level and cognitive function suggested that enhanced TG significantly correlates with executive function deterioration based on second-generation antipsychotic treatment [84]. This result indicates that cognitive deficits in patients with BD may be related to enhanced TG level. Dyslipidemia is an important risk factor for cardiovascular disease in the clinic. One study based on cardiovascular diseases has shown that the shortened life expectancy in patients with BD is closely related to the risk for comorbidities [85] and that enhanced LDL-C could inhibit the working memory task performance of the elderly [86]. Dyslipidemia might alter the GFC in the LIFG, pars triangularis of patients with BD and cause cognitive function and mood disorders. The high cooccurrence of mood disorders and metabolic syndrome suggests that a pathophysiological overlap was well recognized [87].

SVM has been widely used in biomedical diagnosis as an auxiliary method for diagnosis and prediction. The SVM analysis showed that increased GFC values in the left PCu/SOG could be used to distinguish patients with BD from controls with satisfactory accuracy, specificity, and sensitivity of more than 0.7, which is conducive to the establishment of diagnostic indicators. Therefore, we inferred that increased GFC values in the brain area could be used as a potential imaging biomarker to differentiate patients from controls.

The study has limited applicability. First, BD was not classified into different types in the present study. Different types of patients' status might lead to biased results. The sample sizes should be expanded to explore whether or not blood lipids could be utilized as a biomarker for abnormal function in different states of BD. Second, the lipid levels may differ between genders, but no difference was observed in our study. Finally, the confounding effects of smoking, exercise level, or dietary habits on the result were not ruled out.

## 5. Conclusion

The present study is the first to detect voxel-based GFC in BD, which indicates that increased GFC exists in brain regions of the DMN; the LIFG, pars triangularis; and LSOG in patients with BD. Increased GFC values in the LIFG, pars triangularis and left PCu/SOG might be one of the brain functional bases that caused emotional and cognitive dysfunction. The present study provided preliminary evidence that dyslipidemia might be associated with dysfunction in the LIFG, pars triangularis.

## Figures and Tables

**Table 1:** Characteristics of the participants.

	Patients (*n* = 35)	Controls (*n* = 37)	*p* value
Sex (male/female)	11/24	17/20	0.144^a^
Age (years)	20.66 ± 2.51	21.35 ± 3.16	0.593^b^
Years of education (years)	13.91 ± 2.02	14.95 ± 2.11	0.607^b^
HAMD-17	22.14 ± 6.95		
HAMA-14	25.54 ± 8.20		
YRMS	9.26 ± 6.84		
Blood glucose	4.09 ± 1.04		
TG	1.06 ± 0.92		
CHOL	3.88 ± 1.11		
HDL-C	1.27 ± 0.40		
LDL-C	2.41 ± 0.81		
Vocabulary learning	28.36 ± 4.72		
Story retelling	13.64 ± 4.48		
Immediate memory total score	40.76 ± 10.69		
Graphic copy	17.45 ± 2.00		
Line positioning	15.85 ± 3.18		
Visual span total score	32.32 ± 7.21		
Picture named	8.79 ± 0.93		
Verbal fluency test	19.94 ± 4.42		
Verbal function total score	27.88 ± 6.78		
Digit span	14.76 ± 1.79		
Coding test	56.97 ± 10.52		
Attention total score	69.62 ± 16.56		
Vocabulary memory	7.39 ± 1.60		
Vocabulary recognition	19.82 ± 0.58		
Story recall	7.91 ± 2.51		
Figure memory	14.55 ± 3.30		
Delayed memory score	48.21 ± 10.27		
Stroop word	96.30 ± 20.84		
Stroop color	68.42 ± 18.24		
Stroop color-word	41.42 ± 8.81		

^a^A *p* value was obtained by a chi-square test. ^b^The *p* values were obtained by two-sample *t*-tests. HAMD-17: Hamilton Depression Rating Scale-17; HAMA-14: Hamilton Anxiety Scale-14; YRMS: Young Mania Rating Scale.

**Table 2:** Significant differences in GFC values between groups.

Cluster location	Peak (MNI)			Number of voxels	*T* value
	*x*	*y*	*z*		
Patients > controls					
LIFG, pars triangularis	-48	33	18	35	4.1653
Left PCu/SOG	-9	-81	42	53	5.3697

BD: bipolar disorder; GFC: global-brain functional connectivity; LIFG: left inferior frontal gyrus; PCu: precuneus; SOG: superior occipital gyrus; MNI: Montreal Neurological Institute.

**Table 3:** Differentiate the patients from the controls by using the GFC values of a single region with the SVM method.

Brian region	Sensitivity	Specificity	Accuracy
LIFG, pars triangularis	60.00% (21/35)	78.38% (29/37)	69.44% (50/72)
Left PCu/SOG	80.00% (28/35)	75.68% (28/37)	77.78% (56/72)

GFC: global-brain functional connectivity; IFG: inferior frontal gyrus; SVM: support vector machines; LIFG: left inferior frontal gyrus; PCu: precuneus; SOG: superior occipital gyrus.

## Data Availability

The data that support the findings of this study are available from the corresponding author Prof. Wenbin Guo (E-mail: guowenbin76@csu.edu.cn) upon request.

## Supplementary Materials

Supplementary Materials Detailed image acquisition, preprocessing procedures, and the detailed results with each value for male and female are provided in the supplementary material.

## References

[1] Faurholt-Jepsen M., Frost M., Busk J. (2019). Is smartphone-based mood instability associated with stress, quality of life, and functioning in bipolar disorder?. *Bipolar Disorders*.

[2] Di Florio A., Craddock N., van den Bree M. (2014). Alcohol misuse in bipolar disorder. A systematic review and meta-analysis of comorbidity rates. *European Psychiatry*.

[3] Gold A., Otto M., Deckersbach T., Sylvia L., Nierenberg A., Kinrys G. (2018). Substance use comorbidity in bipolar disorder: a qualitative review of treatment strategies and outcomes. *The American Journal on Addictions*.

[4] de Abreu L. N., Nery F. G., Harkavy-Friedman J. M. (2012). Suicide attempts are associated with worse quality of life in patients with bipolar disorder type I. *Comprehensive Psychiatry*.

[5] Miller J., Black D. (2020). Bipolar disorder and suicide: a review. *Current Psychiatry Reports*.

[6] Hirschfeld R. M. (2014). Differential diagnosis of bipolar disorder and major depressive disorder. *Journal of Affective Disorders*.

[7] Huang Y. J., Tsai S. Y., Chung K. H., Chen P. H., Huang S. H., Kuo C. J. (2018). State-dependent alterations of lipid profiles in patients with bipolar disorder. *International Journal of Psychiatry in Medicine*.

[8] Pae C. U., Kim J. J., Lee S. J., Lee C., Paik I. H., Lee C. U. (2004). Aberration of cholesterol level in first-onset bipolar I patients. *Journal of Affective Disorders*.

[9] Wysokiński A., Strzelecki D., Kłoszewska I. (2015). Levels of triglycerides, cholesterol, LDL, HDL and glucose in patients with schizophrenia, unipolar depression and bipolar disorder. *Diabetes and Metabolic Syndrome: Clinical Research and Reviews*.

[10] Cipriani G., Danti S., Carlesi C., Cammisuli D. M., Di Fiorino M. (2017). Bipolar disorder and cognitive dysfunction. *The Journal of Nervous and Mental Disease*.

[11] Martínez-Arán A., Vieta E., Colom F. (2004). Cognitive impairment in euthymic bipolar patients: implications for clinical and functional outcome. *Bipolar Disorders*.

[12] Torrent C., Martinez-Arán A., del Mar Bonnin C. (2012). Long-term outcome of cognitive impairment in bipolar disorder. *The Journal of Clinical Psychiatry*.

[13] Carlson P. J., Singh J. B., Zarate C. A., Drevets W. C., Manji H. K. (2006). Neural circuitry and neuroplasticity in mood disorders: insights for novel therapeutic targets. *NeuroRX*.

[14] Brady R. O., Masters G. A., Mathew I. T. (2016). State dependent cortico-amygdala circuit dysfunction in bipolar disorder. *Journal of Affective Disorders*.

[15] Anticevic A., Brumbaugh M. S., Winkler A. M. (2013). Global prefrontal and fronto-amygdala dysconnectivity in bipolar I disorder with psychosis history. *Biological Psychiatry*.

[16] Rey G., Piguet C., Benders A. (2016). Resting-state functional connectivity of emotion regulation networks in euthymic and non-euthymic bipolar disorder patients. *European Psychiatry*.

[17] Syan S. K., Minuzzi L., Smith M., Allega O. R., Hall G. B., Frey B. N. (2017). Resting state functional connectivity in women with bipolar disorder during clinical remission. *Bipolar Disorders*.

[18] Torrisi S., Moody T. D., Vizueta N. (2013). Differences in resting corticolimbic functional connectivity in bipolar I euthymia. *Bipolar Disorders*.

[19] Syan S. K., Smith M., Frey B. N. (2018). Resting-state functional connectivity in individuals with bipolar disorder during clinical remission: a systematic review. *Journal of Psychiatry & Neuroscience*.

[20] Favre P., Baciu M., Pichat C., Bougerol T., Polosan M. (2014). fMRI evidence for abnormal resting-state functional connectivity in euthymic bipolar patients. *Journal of Affective Disorders*.

[21] Cui X., Liu F., Chen J. (2018). Voxel-wise brain-wide functional connectivity abnormalities in first-episode, drug-naive patients with major depressive disorder. *American Journal of Medical Genetics Part B, Neuropsychiatric Genetics*.

[22] Li T., Wang Q., Zhang J. (2017). Brain-wide analysis of functional connectivity in first-episode and chronic stages of schizophrenia. *Schizophrenia Bulletin*.

[23] Smith S. M., Miller K. L., Salimi-Khorshidi G. (2011). Network modelling methods for FMRI. *NeuroImage*.

[24] Wang Y., Zhong S., Chen G. (2018). Altered cerebellar functional connectivity in remitted bipolar disorder: a resting-state functional magnetic resonance imaging study. *The Australian and New Zealand Journal of Psychiatry*.

[25] Furey T., Christianini N., Duffy N., Bednarski D. W., Schummer M., Hauessler D. (2000). Support vector machine classification and validation of cancer tissue samples using microarray expression data. *Bioinformatics*.

[26] Almansour N. A., Syed H. F., Khayat N. R. (2019). Neural network and support vector machine for the prediction of chronic kidney disease: a comparative study. *Computers in Biology and Medicine*.

[27] Yan C. G., Wang X. D., Zuo X. N., Zang Y. F. (2016). DPABI: data processing & analysis for (resting-state) brain imaging. *Neuroinformatics*.

[28] Buckner R. L., Sepulcre J., Talukdar T. (2009). Cortical hubs revealed by intrinsic functional connectivity: mapping, assessment of stability, and relation to Alzheimer's disease. *The Journal of Neuroscience*.

[29] Donishi T., Terada M., Kaneoke Y. (2017). Effects of gender, digit ratio, and menstrual cycle on intrinsic brain functional connectivity: a whole-brain, voxel-wise exploratory study using simultaneous local and global functional connectivity mapping. *Brain and Behavior*.

[30] Scheinost D., Tokoglu F., Shen X. (2016). Fluctuations in global brain activity are associated with changes in whole-brain connectivity of functional networks. *IEEE Transactions on Biomedical Engineering*.

[31] Farr O. M., Zhang S., Hu S. (2014). The effects of methylphenidate on resting-state striatal, thalamic and global functional connectivity in healthy adults. *The International Journal of Neuropsychopharmacology*.

[32] Pan P., Ou Y., Su Q. (2019). Voxel-based global-brain functional connectivity alterations in first-episode drug-naive patients with somatization disorder. *Journal of Affective Disorders*.

[33] Wang L., Xia M., Li K. (2015). The effects of antidepressant treatment on resting-state functional brain networks in patients with major depressive disorder. *Human Brain Mapping*.

[34] Thompson W. H., Fransson P. (2016). On stabilizing the variance of dynamic functional brain connectivity time series. *Brain Connectivity*.

[35] Kaneoke Y., Donishi T., Iwatani J., Ukai S., Shinosaki K., Terada M. (2012). Variance and autocorrelation of the spontaneous slow brain activity. *PLoS One*.

[36] Cohen J., Cohen P., West S., Aiken L. (2003). *Applied Multiple Regression/Correlation Analysis For The Behavioral Sciences*.

[37] Pan P., Wei S., Ou Y. (2020). Reduced global-brain functional connectivity and its relationship with symptomatic severity in cervical dystonia. *Frontiers in Neurology*.

[38] Ding Y., Ou Y., Su Q. (2019). Enhanced global-brain functional connectivity in the left superior frontal gyrus as a possible endophenotype for schizophrenia. *Frontiers in Neuroscience*.

[39] Li H., Ou Y., Liu F. (2020). Reduced connectivity in anterior cingulate cortex as an early predictor for treatment response in drug-naive, first-episode schizophrenia: a global-brain functional connectivity analysis. *Schizophrenia Research*.

[40] Pan P., Wei S., Li H. (2021). Voxel-wise brain-wide functional connectivity abnormalities in patients with primary blepharospasm at rest. *Neural Plasticity*.

[41] Pan P., Wei S., Ou Y. (2020). Reduced global-brain functional connectivity of the cerebello-thalamo-cortical network in patients with dry eye disease. *Frontiers in Human Neuroscience*.

[42] Song H., Chen L., Gao R. (2017). Automatic schizophrenic discrimination on fNIRS by using complex brain network analysis and SVM. *BMC Medical Informatics and Decision Making*.

[43] Vapnik V. (2000). *The Nature of Statistical Learning Theory*.

[44] Noble W. S. (2006). What is a support vector machine?. *Nature Biotechnology*.

[45] Adewumi A., Owolabi T., Alade I., Olatunji S. (2016). Estimation of physical, mechanical and hydrological properties of permeable concrete using computational intelligence approach. *Applied Soft Computing*.

[46] Chang C.-C., Lin C.-J. (2011). LIBSVM: a library for support vector machines. *ACM Transactions on Intelligent Systems and Technology*.

[47] Nekkaa M., Boughaci D. (2015). A memetic algorithm with support vector machine for feature selection and classification. *Memetic Computing*.

[48] Tozzi A., Peters J. F. (2017). From abstract topology to real thermodynamic brain activity. *Cognitive Neurodynamics*.

[49] Cabeza R. (2002). Hemispheric asymmetry reduction in older adults: the HAROLD model. *Psychology and Aging*.

[50] Guo W., Liu F., Zhang Z. (2015). Increased cerebellar functional connectivity with the default-mode network in unaffected siblings of schizophrenia patients at rest. *Schizophrenia Bulletin*.

[51] Dowlati Y., Herrmann N., Swardfager W. (2010). A meta-analysis of cytokines in major depression. *Biological Psychiatry*.

[52] Liberto C. M., Albrecht P. J., Herx L. M., Yong V. W., Levison S. W. (2004). Pro-regenerative properties of cytokine-activated astrocytes. *Journal of Neurochemistry*.

[53] d'Arbeloff T., Kim M. J., Knodt A., Radtke S., Brigidi B., Hariri A. (2018). Microstructural integrity of a pathway connecting the prefrontal cortex and amygdala moderates the association between cognitive reappraisal and negative emotions. *Emotion*.

[54] Robbins T. W. (2005). Controlling stress: how the brain protects itself from depression. *Nature Neuroscience*.

[55] Zeng L. L., Shen H., Liu L. (2012). Identifying major depression using whole-brain functional connectivity: a multivariate pattern analysis. *Brain*.

[56] Amunts K., Lenzen M., Friederici A. D. (2010). Broca's region: novel organizational principles and multiple receptor mapping. *PLoS Biology*.

[57] Rolls E. T., Cheng W., Du J. (2020). Functional connectivity of the right inferior frontal gyrus and orbitofrontal cortex in depression. *Social Cognitive and Affective Neuroscience*.

[58] Taylor G., Bagby R., Parker J. (1997). *Disorders of Affect Regulation: Alexithymia in Medical and Psychiatric Illness*.

[59] Herold D., Usnich T., Spengler S., Sajonz B., Bauer M., Bermpohl F. (2017). Decreased medial prefrontal cortex activation during self-referential processing in bipolar mania. *Journal of Affective Disorders*.

[60] Yilmaz O., Ates M., Semiz M. (2016). Childhood traumas in patients with bipolar disorder: association with alexithymia and dissociative experiences. *Anatolian Journal of Psychiatry*.

[61] Judd L. L., Schettler P. J., Solomon D. A. (2008). Psychosocial disability and work role function compared across the long-term course of bipolar I, bipolar II and unipolar major depressive disorders. *Journal of Affective Disorders*.

[62] Hobson H., Hogeveen J., Brewer R. (2018). Language and alexithymia: evidence for the role of the inferior frontal gyrus in acquired alexithymia. *Neuropsychologia*.

[63] Batista A. X., Bazán P. R., Conforto A. B. (2019). Resting state functional connectivity and neural correlates of face-name encoding in patients with ischemic vascular lesions with and without the involvement of the left inferior frontal gyrus. *Cortex*.

[64] Favre P., Baciu M., Pichat C. (2013). Modulation of fronto-limbic activity by the psychoeducation in euthymic bipolar patients. A functional MRI study. *Psychiatry Research*.

[65] Bedford N. J., Surguladze S., Giampietro V., Brammer M. J., David A. S. (2012). Self-evaluation in schizophrenia: an fMRI study with implications for the understanding of insight. *BMC Psychiatry*.

[66] Zhang L., Opmeer E. M., Ruhé H. G., Aleman A., van der Meer L. (2015). Brain activation during self- and other-reflection in bipolar disorder with a history of psychosis: comparison to schizophrenia. *NeuroImage: Clinical*.

[67] van der Meer L., Costafreda S., Aleman A., David A. S. (2010). Self-reflection and the brain: a theoretical review and meta-analysis of neuroimaging studies with implications for schizophrenia. *Neuroscience and Biobehavioral Reviews*.

[68] Fink G. R., Markowitsch H. J., Reinkemeier M., Bruckbauer T., Kessler J., Heiss W. D. (1996). Cerebral representation of one's own past: neural networks involved in autobiographical memory. *The Journal of Neuroscience*.

[69] Maddock R. J., Garrett A. S., Buonocore M. H. (2001). Remembering familiar people: the posterior cingulate cortex and autobiographical memory retrieval. *Neuroscience*.

[70] Li R., Utevsky A., Huettel S. (2019). Developmental maturation of the precuneus as a functional core of the default-mode network. *Journal of Cognitive Neuroscience*.

[71] Schmidt S. A., Carpenter-Thompson J., Husain F. T. (2017). Connectivity of precuneus to the default mode and dorsal attention networks: a possible invariant marker of long-term tinnitus. *NeuroImage: Clinical*.

[72] Fransson P., Marrelec G. (2008). The precuneus/posterior cingulate cortex plays a pivotal role in the default mode network: evidence from a partial correlation network analysis. *NeuroImage*.

[73] Uddén J., Snijders T. M., Fisher S. E., Hagoort P. (2017). A common variant of the CNTNAP2 gene is associated with structural variation in the left superior occipital gyrus. *Brain and Language*.

[74] Scheuerecker J., Frodl T., Koutsouleris N. (2007). Cerebral differences in explicit and implicit emotional processing--an fMRI study. *Neuropsychobiology*.

[75] Mei L., Xue G., Chen C., Xue F., Zhang M., Dong Q. (2010). The "visual word form area" is involved in successful memory encoding of both words and faces. *NeuroImage*.

[76] Schraa-Tam C. K. L., Rietdijk W. J. R., Verbeke W. J. M. I. (2012). fMRI activities in the emotional cerebellum: a preference for negative stimuli and goal-directed behavior. *Cerebellum*.

[77] Kim S., Kim Y. W., Jeon H., Im C. H., Lee S. H. (2020). Altered cortical thickness-based individualized structural covariance networks in patients with schizophrenia and bipolar disorder. *Journal of Clinical Medicine*.

[78] Li K., Zhang M., Zhang H. (2020). The spontaneous activity and functional network of the occipital cortex is correlated with state anxiety in healthy adults. *Neuroscience Letters*.

[79] Fagiolini A., Frank E., Scott J. A., Turkin S., Kupfer D. J. (2005). Metabolic syndrome in bipolar disorder: findings from the Bipolar Disorder Center for Pennsylvanians. *Bipolar Disorders*.

[80] Aguglia A., Solano P., Giacomini G. (2019). The association between dyslipidemia and lethality of suicide attempts: a case-control study. *Frontiers in Psychiatry*.

[81] Park Y. M., Lee B. H., Lee S. H. (2014). The association between serum lipid levels, suicide ideation, and central serotonergic activity in patients with major depressive disorder. *Journal of Affective Disorders*.

[82] da Graça Cantarelli M., Nardin P., Buffon A. (2015). Serum triglycerides, but not cholesterol or leptin, are decreased in suicide attempters with mood disorders. *Journal of Affective Disorders*.

[83] Chesney E., Goodwin G. M., Fazel S. (2014). Risks of all-cause and suicide mortality in mental disorders: a meta-review. *World Psychiatry*.

[84] Naiberg M. R., Newton D. F., Collins J. E., Dickstein D. P., Bowie C. R., Goldstein B. I. (2016). Elevated triglycerides are associated with decreased executive function among adolescents with bipolar disorder. *Acta Psychiatrica Scandinavica*.

[85] Vancampfort D., Stubbs B., Mitchell A. J. (2015). Risk of metabolic syndrome and its components in people with schizophrenia and related psychotic disorders, bipolar disorder and major depressive disorder: a systematic review and meta-analysis. *World Psychiatry*.

[86] Meusel L. C., Anderson N. D., Parrott M. D. (2017). Brain function is linked to LDL cholesterol in older adults with cardiovascular risk. *Journal of the American Geriatrics Society*.

[87] Vargas H. O., Nunes S. O. V., Barbosa D. S. (2014). Castelli risk indexes 1 and 2 are higher in major depression but other characteristics of the metabolic syndrome are not specific to mood disorders. *Life Sciences*.

